# A Dialogue in Blank Verse

**Published:** 1908-08-08

**Authors:** 


### A DIALOGUE IN BLANK VERSE

### BETWEEN THE OLD STYLE PRACTITIONER AND THE NEW.

Old Style P.
Sir, these new-fangled ways I must confess
Do, in some measure, shock me! I was born
To ways conventional and manners framed
To suit a state of things that each new change,
Even like the tidal wave that bears us on
From the receding shore, leaves farther off
More indistinct of outline in the dim
And distant haze that old oblivious Time
Spreads o'er each well-known scene!

New Style P. Your language, too,
Is somewhat obsolete! We seldom now
Speak in the measured phrase our fathers loved,
But talk in brief and jerky sentences
Well interspersed with diction of the streets,
Or of the gutter rather! Thus we say
When some fair damsel greets us on our rounds
Not, as of old men said "Good morning, Madam,"
Or "'Tis a fair and lovely time of day,"
But just "So long!" "Pip-Pip!" or "How d'ye do!"
And make a mock of all formality!

Old Style P.
Good gracious, how you shock me! Then your dress
Is—pray, good sir, your pardon!—scarce the thing
That was in my time proper! I was trained
With most punctilious exactitude
To don frock-coat and wear the shining hat
Which fashion then held sacred! When I went
To see my patients, 'twas with fitting state,
With carriage and with harness neat and trim,
With coachman all in faultless livery drest;
But you——

New Style P.
Oh, I, as suits the modern mode,
Affect a jaunty bearing. This lounge suit
Is good enough for me—a cycling cap,
And these tan boots! No coachman drest with care
Goes with me on my rounds—the cycle serves
To take me on my way from street to street,
Or if my patient lives away from town,
I just spin in my motor to the spot,
And shun all loud pretences!

Old Style P. When you call,
I must presume your manner still displays
Some symptoms of formality?

New Style P. Not so!
The master of the house of course I greet
With cheerful friendly feeling! "Well, old boy!
How goes the world with you?" and just "Ta-Ta!"
Again on leaving. 'Tis a simple age,
The age we live in, and punctilious form
Is looked on now as dotage, nothing more!
You with your coat-tails and your shining hat,
Believe me, are as antiquated now,
As is the surgeon Uncle Toby saw
When Tristram Shandy to the world was born!

Old Style P.
Well, sir, you keep your ways and I'll keep mine!
To change my course I must and do decline!
[*Exeunt severally.*]

